# Strengthening provider-initiated testing and counselling in Zimbabwe by deploying supplemental providers: a time series analysis

**DOI:** 10.1186/s12913-019-4169-z

**Published:** 2019-06-03

**Authors:** Aaron F. Bochner, Beth A. Tippett Barr, Batsirai Makunike, Gloria Gonese, Blessing Wazara, Richard Mashapa, Elizabeth Meacham, Ponesai Nyika, Gertrude Ncube, Shirish Balachandra, Ruth Levine, Frances Petracca, Tsitsi Apollo, Ann Downer, Stefan Z. Wiktor

**Affiliations:** 1 International Training and Education Center for Health (I-TECH), Department of Global Health, University of Washington, Seattle, WA USA; 2 Department of Epidemiology, University of Washington, 325 9th Ave, Box 359932, Seattle, WA 98104 USA; 3 U.S. Centers for Disease Control and Prevention, Harare, Zimbabwe; 4 International Training and Education Center for Health (I-TECH), Harare, Zimbabwe; 5 Ministry of Health and Child Care, Harare, Zimbabwe

**Keywords:** HIV testing services, Provider initiated testing and counselling, Human resources, Zimbabwe, Implementation science

## Abstract

**Background:**

Expansion of provider-initiated testing and counselling (PITC) is one strategy to increase accessibility of HIV testing services. Insufficient human resources was identified as a primary barrier to increasing PITC coverage in Zimbabwe. We evaluated if deployment of supplemental PITC providers at public facilities in Zimbabwe was associated with increased numbers of individuals tested and diagnosed with HIV.

**Methods:**

From July 2016 to May 2017, International Training and Education Center for Health (I-TECH) deployed 138 PITC providers to supplement existing ministry healthcare workers offering PITC at 249 facilities. These supplemental providers were assigned to facilities on a weekly basis. Each week, I-TECH providers reported the number of HIV tests and positive diagnoses they performed. Using routine reporting systems, we obtained from each facility the number of clients tested and diagnosed with HIV per month. Including data both before and during the intervention period, and utilizing the weekly variability in placement locations of the supplemental PITC providers, we employed generalized estimating equations to assess if the placement of supplemental PITC providers at a facility was associated with a change in facility outputs.

**Results:**

Supplemental PITC providers performed an average of 62 (SD = 52) HIV tests per week and diagnosed 4.4 (SD = 4.9) individuals with HIV per week. However, using facility reports from the same period, we found that each person-week of PITC provider deployment at a facility was associated with an additional 16.7 (95% CI, 12.2–21.1) individuals tested and an additional 0.9 (95% CI, 0.5–1.2) individuals diagnosed with HIV. We also found that staff placement at clinics was associated with a larger increase in HIV testing than staff placement at polyclinics or hospitals (24.0 vs. 9.8; *p* < 0.001).

**Conclusions:**

This program resulted in increased numbers of individuals tested and diagnosed with HIV. The discrepancy between the average weekly HIV tests conducted by supplemental PITC providers (62) and the increase in facility-level HIV tests associated with one week of PITC provider deployment (16.7) suggests that supplemental PITC providers displaced existing staff who may have been reassigned to fulfil other duties at the facility.

## Background

Countries are striving to reach the ambitious UNAIDS Fast-Track 90–90-90 targets by 2020 [1]. Recent data from population-based studies in several sub-Saharan African countries indicate that achieving the first 90 target – 90% of people living with HIV (PLHIV) knowing their HIV status – is proving the most difficult [2–5]. Globally, there remains a need to identify effective interventions that can expand coverage of HIV testing services (HTS), so more PLHIV know their status, receive antiretroviral therapy, and become virally suppressed, ultimately reducing the rates of HIV transmission, morbidity, and mortality.

The World Health Organization (WHO) has recommended opt-out provider initiated testing and counselling (PITC) in countries with generalized HIV epidemics since 2007 [6]. While routine PITC results in greater uptake of facility-based HTS than patient-initiated voluntary counselling and testing, evaluations of routine program implementation of PITC have generally found that many eligible clients do not receive an HIV test [7, 8]. Lack of human resources has consistently emerged as a barrier to implementation of PITC in resource-limited settings because offering and performing additional HIV tests increases staff workload [9–12].

A population-based survey completed in 2016 in Zimbabwe estimated that 74% of PLHIV knew their HIV status [13]. PITC has been embraced as a testing strategy by the Zimbabwe Ministry of Health and Child Care (MoHCC), with current guidelines prescribing PITC for all clients attending health facilities [14]. In other settings, PITC implementation supported by supplemental staff and adequate supplies of test kits has led to large increases in HIV testing and new diagnoses [15, 16].

To support implementation of PITC, the University of Washington-led International Training and Education Center for Health (I-TECH) in Zimbabwe, in close collaboration with the MoHCC and the U.S. Centers for Disease Control and Prevention (CDC), deployed supplemental PITC providers to public healthcare facilities. PITC providers were supervised within routine MoHCC reporting structures at healthcare facilities, but were expected to dedicate all their time to implementing PITC. We evaluated whether deployment of these providers was associated with an increase in the number of individuals tested and diagnosed with HIV.

## Methods

### I-TECH PITC support

With support from the President’s Emergency Plan for AIDS Relief (PEPFAR) and the CDC, I-TECH has supported HIV testing, care, and treatment services in Zimbabwe since October 2013. In July 2016, I-TECH initiated a program in coordination with the Zimbabwe MoHCC and CDC to hire a cadre of PITC providers. These staff were deployed across the 249 government facilities I-TECH was supporting in 17 districts of Zimbabwe. Hired staff were a combination of nurses and primary counsellors, a lay cadre who receive 9 to 12 months training to provide HIV counselling and testing services.

I-TECH employed the PITC providers, with district MoHCC staff participating in the hiring process for all providers. Hiring terms of reference stated that the PITC providers would be fully dedicated to conducting HIV testing activities. Once hired, providers were supervised by the head MoHCC nurse at each facility (Sisters in Charge). PITC providers worked in a single district, but were assigned to facilities within the district on a weekly basis. I-TECH district staff typically drafted a placement plan for PITC providers each week, based on previous outputs and their general knowledge of facilities, and the MoHCC District Nursing Officer reviewed, modified, and ultimately approved the weekly placements. I-TECH staff had limited ability to monitor the daily activities of the PITC providers because a single district-level I-TECH staff-person was responsible for supporting all PITC providers within the district. With an average of 15 facilities per district, district-level I-TECH staff conducted at least one supervisory visit to all facilities per month. The program ran as described from July 2016 until June 2017, when responsibilities of seconded staff were expanded to include additional activities beyond PITC.

### Data collection and analysis

All public facilities in Zimbabwe reported to the MoHCC monthly on the aggregate number of clients tested and diagnosed with HIV. MoHCC data clerks entered this information into the District Health Information System (DHIS) 2, a national electronic system maintained by the MoHCC. From DHIS2 we obtained monthly statistics from each of the 249 public facilities I-TECH supported.

To measure the level of supplemental PITC provider support at each facility, we calculated the person-weeks that seconded PITC providers worked at each facility per month. The location of seconded PITC providers was monitored through two mechanisms. First, district-level supervisors helped assign providers their weekly placements, which were recorded on spreadsheets submitted to I-TECH’s central office in Harare. Additionally, PITC providers were required at the end of each week to self-report their location and the number of people they tested and diagnosed. Since placements sometimes shifted at the last minute and reports of HIV outputs varied in completeness over time, these two data sources were triangulated to obtain the most complete available data on PITC provider locations. To calculate the total number of supplemental PITC provider person-weeks at each facility per month, weekly placement data were aggregated into monthly totals, with weeks that spanned across months divided into fractions based on the number of weekdays that fell into each month.

We estimated the number of additional HIV tests and new HIV diagnoses associated with one week of supplemental PITC provider deployment by performing bivariate and multivariable analyses using generalized estimating equation models with a Poisson family, identity link, exchangeable correlation matrix, and robust standard errors. Seven months of data from facilities with PITC provider secondments were included in the analysis: October 2016 to February 2017 and April to May 2017. *A priori* decisions were made to exclude July to September 2016, the first three months of the secondment program, due to concerns around completeness and accuracy of the HIV tester placement data. In addition, we excluded March 2017 due to a large MoHCC-led HIV testing campaign that occurred that month in Harare. These analyses also included 12 months of data prior to the start of the PITC provider deployments, July 2015 to June 2016, which provided baseline data on facility-level testing and HIV diagnosis outputs for the 249 facilities. In multivariable models, we adjusted for each month using indicator variables.

Additionally, we evaluated if the outputs of the seconded PITC providers varied when they were placed in different regions or different types of facilities. To do this, we ran multivariable generalized estimating equation models with the addition of a covariate and interaction term for the possible effect modifier, and used the Stata postestimation *lincom* command to calculate estimates of effect and confidence intervals for the different subgroups. We conducted all analyses using Stata version 13.1 (Stata Corporation, College Station, TX).

### Alternative analysis

To address concerns that PITC provider locations were misclassified, we repeated the analysis taking an alternative approach. Rather than using facility-level data, we instead aggregated the total HIV tests and HIV diagnoses from the 249 facilities I-TECH supported per month. As a control group, we aggregated the monthly HIV tests and HIV diagnoses for facilities in 15 districts I-TECH does not support that are located within the same provinces where I-TECH works: Mashonaland East, Mashonaland Central, Mashonaland West, and Matabeleland North. The exposure of interest was the number of seconded PITC providers employed by I-TECH each month, obtained from program records; the control districts always had zero seconded PITC providers. Using a generalized linear model with a Poisson family, identity link, and robust standard errors, we estimated the number of additional HIV tests and diagnoses associated with employment of an additional PITC provider. We transformed the results to present the outputs per person-week of PITC provider employment, consistent with the primary analysis.

## Results

Of the 249 facilities I-TECH supported, 197 were clinics, 15 were polyclinics (large primary care clinics), and 37 were hospitals (Table 1). These facilities were distributed across five provinces. Fifteen percent of sites were in Harare, the only large urban area where supported clinics were located. The number of individuals tested at facilities in the 12 months prior to I-TECH PITC provider secondments varied by facility type: clinics averaged 160 tests per month, hospitals averaged 314 tests per month, and polyclinics averaged 796 tests per month. The number of individuals newly diagnosed with HIV also differed by facility type, with clinics averaging 14 new HIV diagnoses per month, hospitals averaging 29 new diagnoses per month, and polyclinics averaging 73 new diagnoses per month. The overall proportion of clients testing HIV positive in the pre-intervention period was 8.8%.

**Table 1:** Characteristics of the facilities supported by I-TECH before and during deployment of supplemental PITC providers

		Before PITC provider deployment^a^			During supplemental PITC provider deployment^b^			
	Supported facilities (*N* = 249)	Proportion testing HIV-positive	Monthly HIV tests per facility	Monthly HIV diagnoses per facility	Monthly p-w PITC providers per facility	Proportion testing HIV-positive	Monthly HIV tests per facility	Monthly HIV diagnoses per facility
	n (%)	Positive/Tested (%)	Mean (SD)	Mean (SD)	Mean (SD)	Positive/Tested (%)	Mean (SD)	Mean (SD)
Facility type								
Clinic	197 (79%)	31,984/379,147 (8%)	160 (156)	14 (16)	1.5 (1.8)	20,109/288,326 (7%)	209 (172)	15 (21)
Polyclinic	15 (6%)	13,129/143,329 (9%)	796 (442)	73 (42)	1.5 (1.6)	6,811/76,778 (9%)	731 (306)	65 (32)
Hospital	37 (14%)	12,898/139,510 (9%)	314 (250)	29 (25)	2.9 (3.2)	7,605/103,754 (7%)	401 (271)	29 (20)
Province								
Harare	38 (15%)	20,375/214,002 (10%)	469 (403)	45 (33)	1.6 (3.1)	10,909/113,666 (10%)	427 (311)	41 (28)
Mash. Central	53 (21%)	6,613/99,084 (7%)	156 (155)	10 (13)	1.9 (1.9)	3,864/78,627 (5%)	212 (154)	10 (11)
Mash. East	42 (17%)	10,271/102,796 (10%)	204 (195)	20 (26)	1.5 (1.7)	6,373/77,723 (8%)	264 (232)	22 (28)
Mash. West	79 (32%)	16,953/194,046 (9%)	205 (200)	18 (21)	1.9 (1.9)	11,015/163,014 (7%)	295 (255)	20 (27)
Mat. North	37 (15%)	3,796/52,058 (7%)	117 (152)	9 (8)	1.3 (1.9)	2,364/35,828 (7%)	138 (98)	9 (9)
Totals		58,008/661,986 (9%)	222 (254)	19 (25)	1.7 (2.1)	34,525/468,858 (7%)	269 (241)	20 (25)

*p-w* person-week, *Mash* Mashonaland, *Mat* Matabeleland

^a^Time period: July 2015 to June 2016 (12 months)

^b^Time period: October 2016 to May 2017; March 2017 excluded due to MOHCC HIV testing campaign

**Fig. 1:**
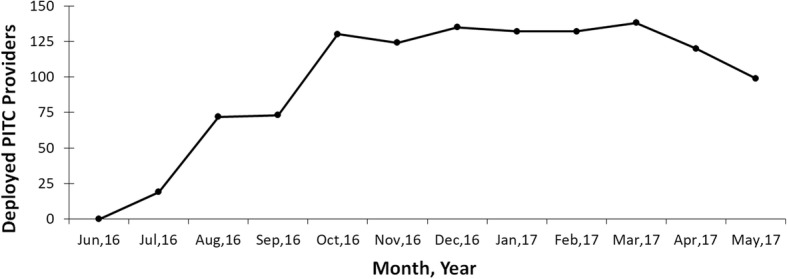
The number of PITC providers deployed to public facilities, June 2016 to May 2017

I-TECH began to hire PITC providers and deploy them to public facilities in July 2016, with a maximum of 138 providers employed in March 2017 (Fig. 1). The number of providers declined in April and May when many resigned due to a large-scale hiring initiative by the MoHCC; seconded providers were signed to short-term contracts while MoHCC positions were permanent. Of the 249 facilities I-TECH supported, all but nine had a PITC provider deployed at the facility for at least one week. On weeks when I-TECH providers were placed at a facility, 89% of the time I-TECH deployed only a single PITC provider at the facility. From October 2016 to May 2017, seconded providers self-reported that they had on average performed 62 (SD = 52) HIV tests per week and diagnosed 4.4 (SD = 4.9) individuals with HIV per week; thus, 7.1% of HIV clients tested by seconded providers were HIV positive.

The total number of HIV tests and new HIV diagnoses reported monthly by each facility was used to estimate the increase in facility outputs attributable to seconded PITC providers. We found that each week a PITC provider was deployed at a facility was associated with an additional 16.7 (95% CI, 12.2–21.1) HIV tests performed and an additional 0.9 (95% CI, 0.5–1.2) individuals newly diagnosed with HIV (Table 2). Thus, 5.4% of the additional individuals tested for HIV associated with PITC provider deployment were HIV positive, lower than the 7.4% (34,525/468,858) of clients who tested positive across the 249 sites during the same 7-month period of PITC provider deployment.

**Table 2:** Change in facility outputs associated with supplemental PITC providers

	Unadjusted model			Adjusted model^a^		
Outcome	Change/p-w	95% CI	*P*-value	Change/p-w	95% CI	*P*-value
HIV tests	21.7	17.0 to 26.5	< 0.001	16.7	12.2 to 21.1	< 0.001
Positive diagnoses	0.7	0.2 to 1.1	0.002	0.9	0.5 to 1.2	< 0.001

*p-w* person-week

^a^Adjusted for calendar time using indicator variables for each month included in the analysis

We also evaluated facility characteristics that could impact the performance of seconded PITC providers (Table 3). Comparing the outputs of PITC providers placed at clinics to those placed at polyclinics or hospitals, we found that providers working at clinics were associated with an additional 24.0 HIV tests per person-week while providers at polyclinics or hospitals were associated an increase of only 9.8 additional tests per person-week (*P*-value < 0.001). HIV diagnoses followed a similar trend with 1.2 additional HIV diagnoses per person-week at clinics compared to 0.4 diagnoses per person-week at polyclinics or hospitals (*P*-value = 0.102). Additionally, we evaluated if PITC provider outputs varied comparing providers placed in urban Harare to providers placed in other primarily rural provinces. PITC providers placed outside Harare yielded a larger number of additional HIV tests compared to providers placed in Harare (18.6 vs. 3.8 tests per person-week; *P*-value = 0.013) but no difference in the number of new HIV diagnoses (0.8 vs. 0.8 diagnoses per person-week; *P*-value = 0.995).

**Table 3:** Exploring facility characteristics that modify the effect of PITC provider placements

	HIV tests^a^			HIV positive diagnoses^a^		
Outcome	Change/p-w	95% CI	*P*-value	Change/p-w	95% CI	*P*-value
Facility type^b^						
Clinic	24.0	18.9 to 29.1	< 0.001	1.2	0.8 to 1.7	< 0.001
Polyclinic/hospital	9.8	4.0 to 15.6	0.001	0.4	−0.5 to 1.3	0.370
Province^c^						
Harare	3.8	−6.6 to 14.3	0.471	0.8	−0.1 to 1.8	0.096
Other	18.6	13.8 to 23.4	< 0.001	0.8	0.4 to 1.3	< 0.001

*p-w* person-week

^a^Adjusted for calendar time using indicator variables for each month included in the analysis

^b^For effect modification of PITC deployment by facility type on HIV testing output, the interaction term *P*-value < 0.001. For effect modification of PITC deployment by facility type on HIV positive diagnoses, the interaction term *P*-value = 0.102

^c^For effect modification of PITC deployment by province on HIV testing output, the interaction term *P*-value = 0.013. For effect modification of PITC deployment by province on HIV positive diagnoses, the interaction term *P*-value = 0.955

### Alternative analysis

To address the concern that misclassification of the placement locations of PITC providers might lead us to underestimate their outputs, we conducted an alternative analysis using data aggregated across all I-TECH supported sites. As a comparison group, we used aggregate outputs from facilities located in 15 surrounding districts where no I-TECH PITC providers were deployed. This analysis found that each person-week of PITC provider deployment was associated with an additional 16.5 (95% CI, 7.8–25.1) HIV tests performed and an additional 0.5 (95% CI, 0.1–1.1) individuals diagnosed with HIV, similar to the results of the primary analysis.

## Discussion

Results indicate that deploying providers to public facilities to support PITC was associated with an increased number of HIV tests and new HIV diagnoses. The challenge of healthcare worker shortages is common across sub-Saharan African countries, including in Zimbabwe, one of 100 countries where the density of doctors, nurses, and midwives is below the threshold estimated to achieve relatively high coverage for essential health interventions [17, 18]. Additionally, in Zimbabwe a government freeze on hiring nurses was in place from 2012 to 2017, a challenge faced in other settings [19, 20]. Though the MoHCC had put policies in place that mandated PITC for all clients, healthcare worker shortages contributed to an inability to fully implement these policies.

In the context of large Global Health Initiatives such as PEPFAR, the Global Fund Against AIDS, Tuberculosis, and Malaria, and Gavi, healthcare worker secondment to support public healthcare facilities has become a common practice [20–23]. However, quantitative evaluations of the impact of such initiatives have been lacking. Funding from Global Health Initiatives often comes with targets to achieve a number of disease-specific outputs, and both funders and implementing organizations often recognize that human resources are a major constraint when attempting to scale-up activities [19, 21, 24]. The secondment program described in this manuscript was developed within this context, and I-TECH was expected to quickly expand HIV testing services.

By supplementing available human resources through secondments, the program increased the number of individuals tested and diagnosed with HIV. This evaluation estimated that supplemental PITC providers increased facility HIV testing outputs by 16.7 tests per person-week with 0.9 additional HIV diagnoses per person-week. At the height of the program, with 138 PITC providers deployed, our results estimate that the secondment program tested an additional 9987 clients per month and diagnosed an additional 538 individuals per month with HIV. Our estimate that 5.4% of the additional clients tested for HIV due to the secondment program were HIV-positive is lower than the 7.4% positivity among all clients tested during the same period, suggesting that pre-existing providers were testing the highest risk clients and that the expansion of PITC led to testing of a larger but lower-risk population.

There is no consensus on the number of clients that a single individual should be able to counsel and test in a workday. A busy HIV testing campaign in Tanzania reported that staff tested 15 clients per day [25], while Kenya’s Ministry of Health suggested that staff should test a maximum of 10 clients per day [26]. Using any benchmark, the additional 16.7 tests performed per person-week of PITC provider deployment is less than one would expect from an HIV tester working at a busy clinic. This result stands in contrast to the average 62 tests per week that PITC providers reported conducting through our routine program monitoring system. We believe the most likely explanation for this discrepancy is that I-TECH PITC providers displaced existing staff who had been conducting HIV testing at facilities. Prior to I-TECH’s PITC provider deployments, MoHCC had at least one trained HIV tester working at each facility, with larger facilities having up to three testers. As described above, public healthcare facilities in Zimbabwe are understaffed, affecting the delivery of all health services. We suspect that when clinics received a new staff member dedicated to conducting HIV testing, they reassigned staff who had been conducting HIV testing to provide other services at the facility. Our data suggest that of the 62 tests done weekly by seconded providers, 17 (27%) were additive while the remaining 45 (73%) were displacement and would have been done by existing staff had the seconded tester not been at the facility. It is important to note that we do not believe this displacement occurred because PITC was saturated and there were no additional clients available to test. Reports from site visits conducted throughout the period of tester secondment consistently found that PITC was not reaching saturation.

We also believe that displacement likely explains the effect modification we observed. Providers placed at polyclinics, hospitals, or in Harare were associated with smaller increases in the number of clients tested for HIV. Yet providers working at polyclinics or hospitals self-reported performing slightly more HIV tests per week than providers working at smaller clinics (66 compared to 60 tests per week). These larger, urban facilities were more likely to have staff dedicated to conducting PITC prior to I-TECH tester secondments and were more likely to experience a larger displacement effect when an I-TECH tester was deployed. If I-TECH PITC providers did displace existing staff, freeing those staff to address other health needs, the program likely produced other health benefits for clients. However, those activities are outside the original objectives of the secondment program and measuring those benefits is beyond the scope of this evaluation.

This analysis had several limitations. The most significant limitation was our inability to verify the weekly PITC provider placement locations. Misclassification of provider locations would result in under-estimation of the number of additional tests and new diagnoses associated with PITC provider deployment. We addressed this limitation by conducting an alternative analysis pooling tester placements and outputs across the 249 facilities I-TECH supports, which produced similar estimates to the primary results. A second limitation is that PITC providers may have over-reported the number of HIV tests and diagnoses they conducted, which would lead us to over-estimate the amount of displacement that occurred. Though some over-reporting is possible, we believe it is unlikely to account for the majority of displacement we observed. PITC providers never received a financial incentive to increase outputs that might motivate over-reporting. Additionally, the 62 tests per 5-day workweek represent a plausible output in the context of a busy clinic with excess clients eligible for HIV testing. This is also consistent with what I-TECH staff observed anecdotally during supervisory site visits.

Strengths of this analysis include the large number of facilities that participated in the secondment program and inclusion of both urban and rural settings, which extends the generalizability of our findings. Additionally, the inclusion of 12 months of data prior to the HIV tester secondments and seven months of data from the intervention period enabled us to estimate our primary outcomes with good precision. Lastly, the fact that the location of HIV tester placements fluctuated during the implementation period allowed us to adjust for any secular trends or seasonal variability that affected the number of individuals tested and diagnosed for HIV at I-TECH supported facilities.

To further increase coverage of PITC, I-TECH will need to engage MoHCC at the facility, district, and provincial levels to reduce staff displacement. One strategy I-TECH is now testing to reduce displacement is to concentrate staff deployments at high-volume sites where PITC coverage is known to be lowest, allowing I-TECH staff to more closely monitor implementation at the facility level. I-TECH will evaluate this new strategy in the future.

## Conclusions

This evaluation demonstrates that deploying additional PITC providers successfully increased the number of individuals tested and diagnosed with HIV. However, our results suggest that a majority of work done by supplemental PITC providers displaced existing MoHCC staff. In healthcare settings such as Zimbabwe with severe shortages in human resources exacerbated by a five-year hiring freeze, some amount of staff displacement may be unavoidable; facility managers face ongoing challenges allocating sufficient staff to provide other urgently needed healthcare services. Within this context, scaling up a single healthcare program in isolation is a challenge, and future human resource interventions should be designed to limit the impact of staff displacement or target a broader range of patient needs.

## Data Availability

For parties interested in obtaining datasets used for this evaluation, all requests have to be processed and approved by the Zimbabwe Ministry of Health and Child Care. To submit a request, readers can email the Deputy Director for HIV/AIDS and STIs at tsitsiapollo2@gmail.com.

## Abbreviations

CDC: U.S. Centers for Disease Control and Prevention
CI: Confidence inverval
HTS: HIV testing services
I-TECH: International Training and Education Center for Health
MoHCC: Ministry of Health and Child Care
PEPFAR: President’s Emergency Plan for AIDS Relief
PITC: Provider-initiated testing and counselling
PLHIV: People living with HIV
SD: Standard deviation
WHO: World Health Organization
